# Overexpression of *HSPA1A* enhances the osteogenic differentiation of bone marrow mesenchymal stem cells via activation of the Wnt/β-catenin signaling pathway

**DOI:** 10.1038/srep27622

**Published:** 2016-06-09

**Authors:** Wei Zhang, Deting Xue, Houfa Yin, Shengdong Wang, Chao Li, Erman Chen, Dongcai Hu, Yiqing Tao, Jiawei Yu, Qiang Zheng, Xiang Gao, Zhijun Pan

**Affiliations:** 1 Department of Orthopedics, Second Affiliated Hospital, School of Medicine, Zhejiang University, 310009, Hangzhou, China; 2 Eye Center, Second Affiliated Hospital, School of Medicine, Zhejiang University, 310009, Hangzhou, China; 3 Department of Surgery, Second Affiliated Hospital of Zhejiang University School of Medicine, 310009, Hangzhou, Zhejiang Province, China; 4 Department of Orthopedics, Zhuji People’s Hospital of Zhejiang Province, Zhuji, Zhejiang Province, China

## Abstract

*HSPA1A*, which encodes cognate heat shock protein 70, plays important roles in various cellular metabolic pathways. To investigate its effects on osteogenic differentiation of bone marrow mesenchymal stem cells (BMSCs), its expression level was compared between undifferentiated and differentiated BMSCs. Rat *HSPA1A* overexpression in BMSCs increased osteoblast-specific gene expression, alkaline phosphatase activity, and mineral deposition *in vitro*. Moreover, it upregulated β-catenin and downregulated *DKK1* and *SOST*. The enhanced osteogenesis due to *HSPA1A* overexpression was partly rescued by a Wnt/β-catenin inhibitor. Additionally, using a rat tibial fracture model, a sheet of *HSPA1A*-overexpressing BMSCs improved bone fracture healing, as determined by imaging and histological analysis. Taken together, these findings suggest that *HSPA1A* overexpression enhances osteogenic differentiation of BMSCs, partly through Wnt/β-catenin.

Mesenchymal stem cells (MSCs) retain their self-renewal capability and have the potential to differentiate into a variety of cell types^1^; thus, they have emerged as the most promising candidate for tissue repair. It is essential to obtain a deeper understanding of the osteogenic differentiation of MSCs for clinical applications in skeletal regenerative medicine, as well as of their physical and pathological mechanisms in bone metabolism^2^. Thus, identifying the genetic factors involved in osteogenic differentiation of MSCs has become a very important area of research.

Heat shock protein (HSP) 70 family is evolutionarily highly conserved molecular chaperone stabilizes existing proteins against aggregation and mediates the folding of newly translated proteins^3^,^4^. Although it is transiently expressed in cells following heat shock or other stresses, it is also constitutively expressed in some tissues, correlating with senescence and chemoresistance^5^. The constitutive member is often referred to as cognate heat shock protein (HSC70), which is encoded by Heat shock protein family A member 1A (*HSPA1A*)^3^,^6^. It could be a post-transcriptional regulator of gene expression that binds and stabilizes select mRNAs containing AU-rich elements^7^. Increasing evidence has revealed that the appropriate heat stress promotes osteogenic differentiation of MSCs or osteoprogenitor cells^8^,^9^,^10^, which induces the expression of HSPs, including HSC70. In addition, HSC70 is believed to play an important role in bone formation, since it is highly expressed in new bone-generating areas^11^,^12^. These findings indicate that intracellular expression of *HSPA1A* might play a positive role in osteogenic differentiation of MSCs.

Wnt/β-catenin signaling is a crucial regulator of MSCs^13^ and plays an essential role in osteogenic differentiation^14^,^15^. Disruption of β-catenin leads to extensive bone marrow adiposity and low bone mass^16^. Wnt ligands bind to Frizzled and LRP5/6 receptors and induce stabilization of cytoplasmic β-catenin by inhibiting GSK3β^15^. β-catenin accumulates in the cytoplasm and travels to the nucleus, where it engages the N-terminus of DNA-binding proteins of the Tcf/Lef family^17^, thereby affecting target gene transcription. The binding of dickkopf-1 (DKK1) and sclerostin (SOST) to LRP5/6 or Frizzled coreceptor was shown to block Wnt/β-catenin signaling^18^,^19^. Interestingly, David *et al*. revealed crosstalk between HSC70 and Wnt/β-catenin signaling; that is, *HSPA1A* is upregulated in response to Wnt activation in stem cells^20^.

In this study, we found the endogenous expression of *HSPA1A* increased during the process of osteogenic differentiation. Accordingly, we hypothesize that upregulated expression of *HSPA1A* could promote osteogenic differentiation of MSCs via Wnt/β-catenin signaling. by assessing the expression levels of specific genes and calcium deposition. We revealed that *HSPA1A* overexpression enhanced osteogenic differentiation of rat bone marrow MSCs (rBMSCs) partly via the Wnt/β-catenin signaling pathway *in vitro*. Moreover, using a rat tibial fracture model, we found that a sheet of rBMSCs overexpressing *HSPA1A* improved bone fracture healing *in vivo*.

## Materials and Methods

### Cells and reagents

rBMSCs were purchased from Cyagen Biosciences (Guangzhou, China). These cells can differentiate into osteoblasts, adipoblasts, and chondrocytes under specific inductive conditions. Adherent cells were trypsinized and passaged after reaching 80% confluence. Cells from passages 3–9 were used in subsequent experiments.

Recombinant DKK1 was purchased from PeproTech (Rocky Hill, NJ, USA). In accordance with a previous study, the applied concentration of DKK1 was 0.5 μg/mL^21^.

### Lentiviral packaging and cell infection

Lentivirus overexpressing *HSPA1A* (lenti-*HSPA1A*, pLV[Exp]-Puro-CMV >rat-*HSPA1A* [NM_031971.2]: IRES: EGFP) particles and lentiviral GFP particles (lenti-control, pLV[Exp]-Puro-CMV >EGFP) were prepared by Cyagen Biosciences. The overexpressing *HSPA1A* lentivirus particles details are specified in Supplemental material 1. The lentiviral GFP particles were used as control group in this study.

For infections, rBMSCs were incubated with lentiviral particles and polybrene (5 μg/mL) in growth medium. After 5–6 h, the infection medium was discarded. After 3 days, the cells were screened using puromycin (4 μg/mL; Sigma, Shanghai, China) and then passaged for use in subsequent experiments. The expression of *HSPA1A* was quantified by quantitative real-time polymerase chain reaction (qPCR) and Western blot analyses.

### Cell Counting Kit-8 (CCK-8)

To assess the effect of *HSPA1A* overexpression on the proliferation of rBMSCs, the cells were seeded into a 96-well plate (5000/well) and allowed to adhere for 24 h. After 24 h, the medium was removed, and the cells were treated with 10% CCK-8 (Dojindo, Kumamoto, Japan) in 100 μL low-sugar Dulbecco’s modified Eagle’s medium (L-DMEM) without fetal bovine serum (FBS) for 2 h at 37 °C. Absorbance at 450 nm, which is directly proportional to cell proliferation, was measured using a microplate reader (ELX808; BioTek, Winooski, VT, USA).

### Osteogenic differentiation protocol

MSCs were cultured in growth medium [L-DMEM; 10% FBS (1495527; Gibco, Waltham, MA, USA) and 100 IU/mL penicillin/streptomycin] in 6- or 12-well cell culture plates (Corning, Shanghai, China), at a density of 3 × 10^4^/cm^2^, and incubated for 48 h at 37 °C under 5% CO_2_. The cells were subsequently cultured in osteogenic induction medium (L-DMEM with 10% FBS, 100 IU/mL penicillin/streptomycin, 100 nM dexamethasone, 0.2 mM ascorbic acid, and 10 mM β-glycerophosphate). The cells were maintained by the addition of fresh osteogenic induction medium every 2–3 days.

### Measurement of alkaline phosphatase (ALP) activity

For the measurement of ALP activity, cells were lysed in radioimmunoprecipitation assay (RIPA) lysis buffer (Beyotime, Shanghai, China), and the lysate (10 μL) was incubated with 90 μL fresh solution containing p-nitrophenyl phosphate substrate at 37 °C for 30–60 min. The reaction was stopped by the addition of 0.5 N NaOH (100 μL), and the absorbance was measured at 405 nm using a microplate reader (ELX808; BioTek). The total protein concentration was measured using a BCA protein assay kit (KeyGen BioTECH, Nanjing, China). The relative ALP activity is expressed as the percentage change in optical density (OD) per unit time per milligram protein: (OD/15 min/mg protein) ×100.

### Alizarin red staining (ARS)

After the induction of osteogenic differentiation, mineral deposition was assessed by ARS (Cyagen Biosciences). Cells were fixed in 4% paraformaldehyde (Sangon Biotech, Shanghai, China) for 15 min at room temperature and then washed with distilled water. A 1% solution of alizarin red was added and incubated for 30 min at room temperature, followed by rinsing with distilled water. The solution was collected, and 200 μL were plated on 96-well plates, which were read at 560 nm using a microplate reader (ELX808; BioTek). The readings were normalized to the total protein concentration.

### Immunofluorescence

Cells were cultured in induction medium in a 12-well plate, and RUNX2, COL1A1, and β-catenin were detected using a fluorescence microscope (EU5888; Leica, Wetzlar, Germany). Briefly, cells were fixed in 4% paraformaldehyde for 15 min at room temperature, permeabilized, and blocked for 30 min in 0.05% Triton X-100 and 2% bovine serum albumin. Fixed cells were washed and incubated overnight with anti-RUNX2 (1:1600; Cell Signaling Technology, Shanghai, China), COL1A1 (1:100; Santa Cruz Biotechnology, Shanghai, China), or β-catenin (1:100; Cell Signaling Technology). Cells were incubated with a fluorescence-conjugated secondary antibody (Beyotime) for 120 min, and nuclei were stained with 4′,6-diamidino-2-phenylindole (KeyGen Biotech, Nanjing, China) for 2 min. Samples were observed under a fluorescence microscope (Leica).

### RNA isolation and qPCR

Total cellular RNA was isolated using RNAiso reagent (Takara, Dalian, China) and quantified by measuring the absorbance at 260 nm (NanoDrop 2000; Thermo Fisher Scientific, Waltham, MA, USA). Total RNA (≤1000 ng) was reverse-transcribed into cDNA in a reaction volume of 20 μL using the Double-Strand cDNA Synthesis Kit (Takara, Dalian, China). One microliter of cDNA was used as the template for the qPCR reaction. All gene transcripts were quantified by qPCR using the Power SYBR® Green PCR Master Mix (Takara) on the ABI StepOnePlus System (Applied Biosystems, Warrington, UK). The mRNAs of the target genes and the housekeeping gene (18S) were quantified in separate tubes. All primers were synthesized by Sangon Biotech (Shanghai, China). The primer sequences used are shown in Table 1. The cycle conditions were as follows: 95 °C for 30 s and then 40 cycles of 95 °C for 5 s and 60 °C for 30 s. The relative target gene expression levels were calculated using the 2^−△△Ct^ method.

### Western blot analysis

Cells were lysed in RIPA lysis buffer supplemented with a proteasome inhibitor (Beyotime). Total proteins were separated by 10% sodium dodecyl sulfate polyacrylamide gel electrophoresis and then transferred to a polyvinylidene fluoride membrane (Millipore, Shanghai, China). After blocking in 5% non-fat milk for 2 h, the membranes were incubated overnight at 4 °C with antibodies specific to glyceraldehyde-3-phosphate dehydrogenase (GAPDH) (1:1500, Cell Signaling Technology), *HSPA1A* (1:1000; Cell Signaling Technology), Osteocalcin (OCN) (1:1000; abcam, Shanghai, China), RUNX2 (1:1600), COL1A1 (1:1000), or β-catenin (1:1000). Horseradish peroxidase (HRP)-conjugated goat anti-rabbit IgG (1:1500; Cell Signaling Technology) was applied as a secondary antibody for 2 h at room temperature. The immunoreactive bands were detected using an enhanced chemiluminescent detection reagent (Millipore, Shanghai, China). Signal intensity was measured using a Bio-Rad XRS chemiluminescence detection system (Bio-Rad, Hercules, CA, USA).

### Cell sheet preparation

A cell sheet was fabricated as we reported previously^22^. Briefly, confluent cells in flasks at 1 × 10^5^/cm^2^ were cultured in L-DMEM with the addition of vitamin C (20 μg/mL)^23^ for 2 weeks until a sheet of rBMSCs formed and could be detached intact from the substratum using a cell scraper (Supplemental material 2).

### *In vivo* evaluation in animals

All Sprague Dawley (SD) rats were supplied by the Academy of Medical Sciences of Zhejiang province. All animal experiments were performed in accordance with the Animal Care and Use Committee guidelines of Zhejiang province. All experimental procedures were approved by the Institutional Animal Care and Use Committee at Zhejiang university.

All surgical procedures were performed by two experienced traumatic orthopedic surgeons (Zhijun Pan and Deting Xue). Fractures were generated in 8-week-old male SD rats (weighing approximately 200 g). The rats were anesthetized with 0.3% pentobarbital sodium (Sigma) intraperitoneally at 30 mg/kg body weight. The fracture model was established as reported previously^24^,^25^,^26^. After anesthesia, an incision was made below the knee. A transverse osteotomy from front to back was generated at the tibia (above the fibular junction) using an oscillating mini saw. An intramedullary needle (1.2-mm-diameter stainless steel syringe needle) was inserted inside the medullary canal of the tibia for fixation. The gap was 1.0 mm (Supplemental material 3). The 15 fractures in 15 rats were randomized into three groups. In the blank group (n = 5), nothing was grafted into the fracture site; in the lenti-control group (n = 5), a sheet of lenti-control rBMSCs was wrapped around the fracture site; and in the lenti-*HSPA1A* group (n = 5), a sheet of rBMSCs overexpressing *HSPA1A* was wrapped around the fracture site.

### Radiographic analysis and micro-computed tomography (CT) evaluation

Animals were euthanized 8 weeks after surgery, and samples were collected. Radiographs were taken using a dual-track molybdenum/rhodium^+^ Mo target mammography machine (22 KV, 250 mAS; GE, Fairfield, CT, USA) 8 weeks after surgery to evaluate callus formation and bridging bone formation at the fracture sites. All samples were scanned for bone formation using a μCT 100 imaging system (Scanco Medical, Brüttisellen, Switzerland) with the following scan parameters: X-ray energy setting of 70 kVp, 1024 reconstruction matrix, slice thickness of 0.0148 mm, and integration time of 300 ms. The bone volume fraction (BV/TV) including the area of bone formation was calculated by three-dimensional standard microstructural analysis^27^.

### Histological evaluation

The samples were fixed using 4% paraformaldehyde for 72 h at room temperature and then decalcified using 10% ethylenediaminetetraacetic acid (Sigma), with a solution change once a week for more than 8 weeks, before embedding in paraffin. Serial sections of 3 mm thickness were cut and mounted on polylysine-coated slides. Hematoxylin and eosin, Safranin O, Masson, and immunofluorescence staining of COL1A1 were performed separately on consecutive tissue sections in accordance with previous studies^24^,^28^, and images were obtained using a microscope.

### Statistical analysis

Statistical analysis was performed using SPSS 17.0 software (IBM, Armonk, NY, USA). All experiments were performed at least in triplicate, and the data are presented as means ± standard deviation. Statistical significance was determined using a two-tailed Student’s *t*-test when comparing two groups, and one-way ANOVA followed by Bonferroni’s *post hoc* test when comparing more than two groups. A *P* value of 0.05 or less was considered to represent a statistically significant difference.

## Results

### Endogenous *HSPA1A* expression

**Figure 1:**
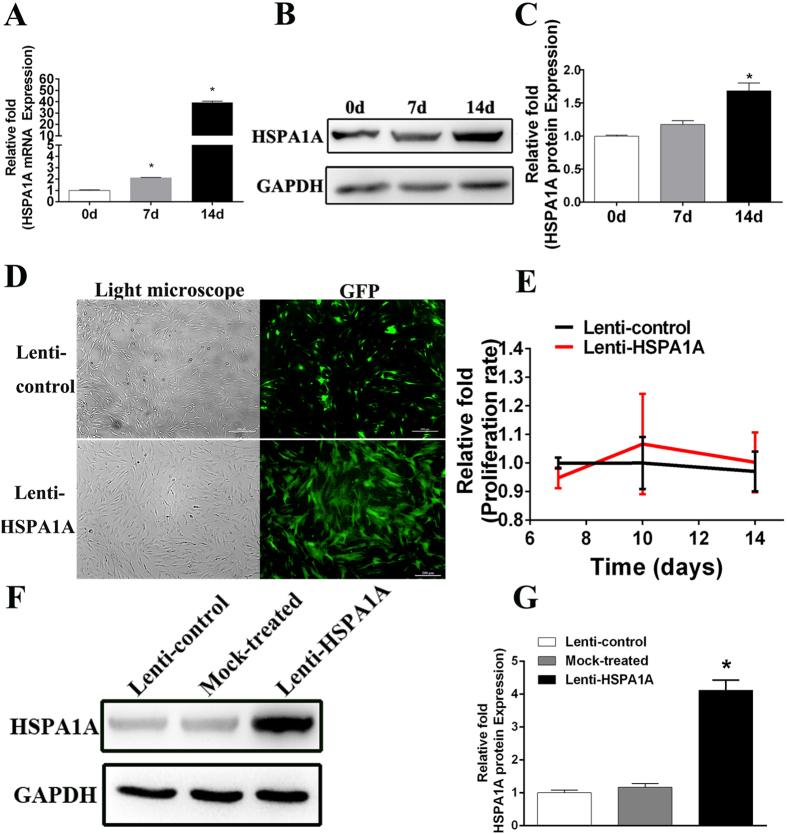
Endogenous *HSPA1A* expression and the construction of *HSPA1A*-overexpressing rBMSCs and lenti-control rBMSCs. (**A**) The endogenous expression of *HSPA1A* mRNA was determined by qPCR at days 0, 7, and 14 of osteogenic differentiation. (**B,C**) The endogenous expression of *HSPA1A* protein was determined by Western blot analysis at days 0, 7, and 14 of osteogenic differentiation. (**D**) rBMSCs after lentiviral transfection and puromycin screening were observed under a normal microscope and a fluorescence microscope. (**E**) The proliferation rate of rBMSCs was not significantly affected by *HSPA1A* overexpression. (**F,G**) Protein levels of *HSPA1A* were determined by Western blot analysis among the lenti-*HSPA1A*, mock treated group, and lenti-control group. The mRNA expression levels were normalized to that of 18S ribosomal RNA. The data are expressed as means ± standard deviation, n = 3, *P < 0.05 vs. the lenti-control group.

**Figure 2:**
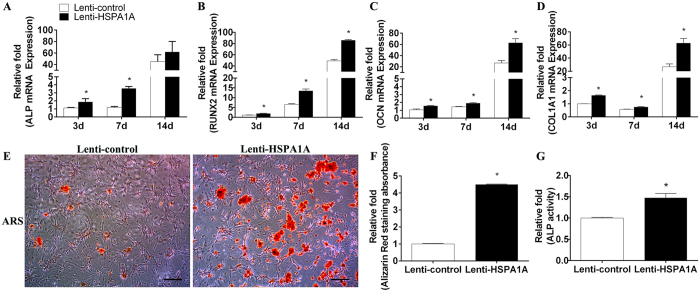
The overexpression of *HSPA1A* promoted osteogenic differentiation of rBMSCs. (**A**) The expression of ALP mRNA was determined by qPCR at days 3, 7, and 14 of osteogenic differentiation. (**B**) The expression of RUNX2 mRNA (**C**) The expression of OCN mRNA (**D**) The expression of COL1A1 mRNA (**E**) Alizarin red staining in the lenti-control and lenti-*HSPA1A* overexpression groups at day 28 of osteogenic differentiation. Scale bar = 200 μm (**F**) Alizarin red staining area determined by measuring the absorbance at 560 nm. (**G**) ALP activity in the control and *HSPA1A* overexpression groups at day 14 of osteogenic differentiation. The mRNA expression levels were normalized to that of 18S ribosomal RNA. The data are expressed as means ± standard deviation of three independent experiments, and one of three independent experiments is shown. *P < 0.05 vs. the lenti-control group.

To determine the expression levels of *HSPA1A* associated with osteogenic differentiation of MSCs, we examined *HSPA1A* endogenous expression in rBMSCs at days 0, 7, and 14 during the process of osteogenic differentiation. Compared with undifferentiated rBMSCs, the mRNA expression of *HSPA1A* increased significantly at days 7 and 14 during osteogenic differentiation (Fig. 1A). In addition, the protenin expression of *HSPA1A* also increased significantly at day 14 during osteogenic differentiation (Fig. 2B,C).

### The establishment of *HSPA1A* overexpression in rBMSCs

To clarify the role of *HSPA1A* during osteogenic differentiation, a lentiviral vector system (Supplemental material 1) was used efficiently to overexpress *HSPA1A* in >80% of third-generation rBMSCs, which was quantified by evaluating the ratio of green fluorescent protein (GFP)-positive cells to the total cell number (Fig. 1D). *HSPA1A* expression was quantified by Western blot analyses 1 week after infection and screening. Compared with those in the lenti-control group and mock treated group (without virus), *HSPA1A* were overexpressed 4.12-fold in the lenti-*HSPA1A* group (Fig. 1F,G).

### *HSPA1A* overexpression did not affect rBMSC proliferation

To determine whether *HSPA1A* overexpression influences the proliferation of rBMSCs, CCK8 detection was performed. The effects of *HSPA1A* overexpression on rBMSC proliferation at days 7, 10, and 14 after infection following culture in normal growth medium are shown in Fig. 1E. No significant difference was detected in the cell proliferation rate between *HSPA1A*-overexpressing and non-overexpressing rBMSCs.

### *HSPA1A* overexpression increased the levels of osteo-specific genes and proteins

To assess the role of *HSPA1A* overexpression in osteogenic differentiation, the levels of osteo-specific genes and proteins, including ALP, RUNX2, osteocalcin (OCN), and COL1A1, were detected by qPCR and Western blot analyses. qPCR analysis revealed that ALP, RUNX2, OCN, and COL1A1 mRNA levels were significantly higher in rBMSCs overexpressing *HSPA1A* at days 3, 7, and 14 than in the non-overexpressing rBMSCs (*P* < 0.05, Fig. 2A–D).

**Figure 3:**
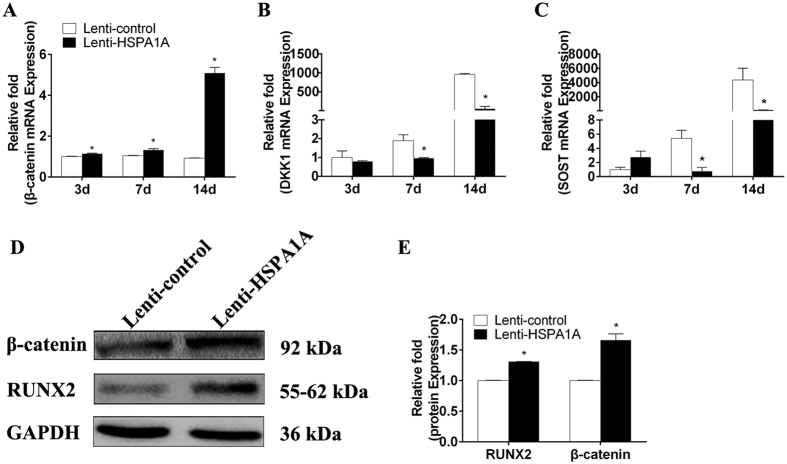
The overexpression of *HSPA1A* upregulated the Wnt/β-catenin signaling pathway during rBMSC osteogenesis. (**A**) The expression of β-catenin mRNA was determined by qPCR at days 3, 7, and 14 of osteogenic differentiation. (**B**) The expression of DKK1 mRNA (**C**) The expression of SOST mRNA (**D**,**E**) The expression of RUNX2 and β-catenin proteins was determined by Western blot analysis after osteogenic differentiation for 7 days. The mRNA expression levels were normalized to that of 18S ribosomal RNA. The protein expression levels were normalized to that of GAPDH. The data are expressed as means ± standard deviation of three independent experiments, and one of three independent experiments is shown. *P < 0.05 vs. the lenti-control group.

Western blot analysis revealed a higher level of RUNX2 protein expression in rBMSCs overexpressing *HSPA1A* than in the non-overexpressing cells (Fig. 3D).

**Figure 4:**
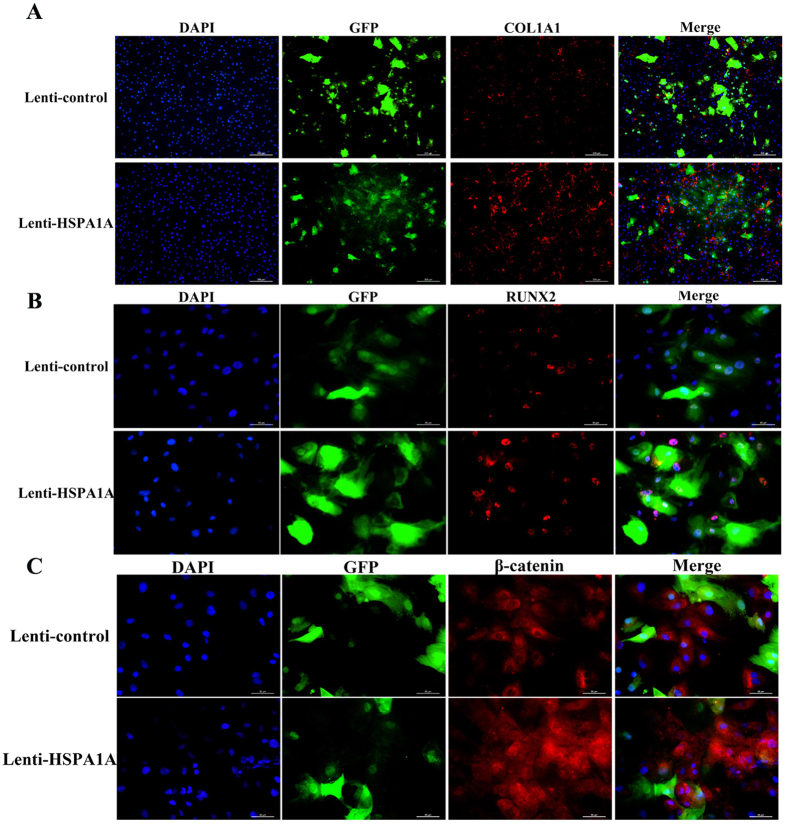
Immunofluorescence staining showed that the protein levels of COL1A1, RUNX2, and β-catenin were up-regulated by *HSPA1A* overexpression. (**A**) The level of COL1A1 protein (red) at day 7 of osteogenic differentiation. The nuclei are counterstained with DAPI (blue). Scale bar = 200 μm. (**B**) The level of RUNX2 protein (red) at day 7 of osteogenic differentiation. The nuclei were counterstained with DAPI (blue). Scale bar = 50 μm. (**C**) The level of β-catenin protein at day 7 of osteogenic differentiation. The nuclei were counterstained with DAPI (blue). Scale bar = 50 μm.

We also used immunofluorescence to confirm the expression of RUNX2 and COL1A1 proteins and showed that these protein expression levels increased at day 7 in *HSPA1A*-overexpressing cells (Fig. 4A,B).

### *HSPA1A* overexpression enhanced ALP activity and calcium deposit formation

We evaluated ALP activity, an early marker of osteogenesis, at day 14 during osteogenic differentiation. Compared with the non-overexpression group, higher ALP activity was observed in the *HSPA1A* overexpression group (*P* < 0.05, Fig. 2G). Calcium deposits were also examined by ARS, and the staining areas were quantified by measuring the absorbance at 560 nm. More calcium deposits appeared in the *HSPA1A*-overexpressing than in the non-overexpressing rBMSCs at day 28 (Fig. 2E,F).

### *HSPA1A* overexpression activated the Wnt/β-catenin signaling pathway

To confirm the above findings suggesting that Wnt/β-catenin signaling is involved in the observed phenomena, the expression of β-catenin was determined by qPCR, Western blot analysis, and immunofluorescence. The expression of *DKK1* and *SOST* was also detected by qPCR. The results of qPCR and Western blot analyses demonstrated higher expression of β-catenin in the rBMSCs overexpressing *HSPA1A* (Fig. 3A,D,E). Compared with those in the non-overexpression group, *DKK1* and *SOST* levels were significantly reduced in the rBMSCs overexpressing *HSPA1A* (Fig. 3B,C). Moreover, using immunofluorescence, we found lower levels of β-catenin accumulation, the majority of which was in the cytoplasm in the non-overexpression group (Fig. 4C).

### The increased osteogenic differentiation of rBMSCs due to *HSPA1A* overexpression was partially rescued by the addition of a Wnt/β-catenin signaling inhibitor (DKK1)

**Figure 5:**
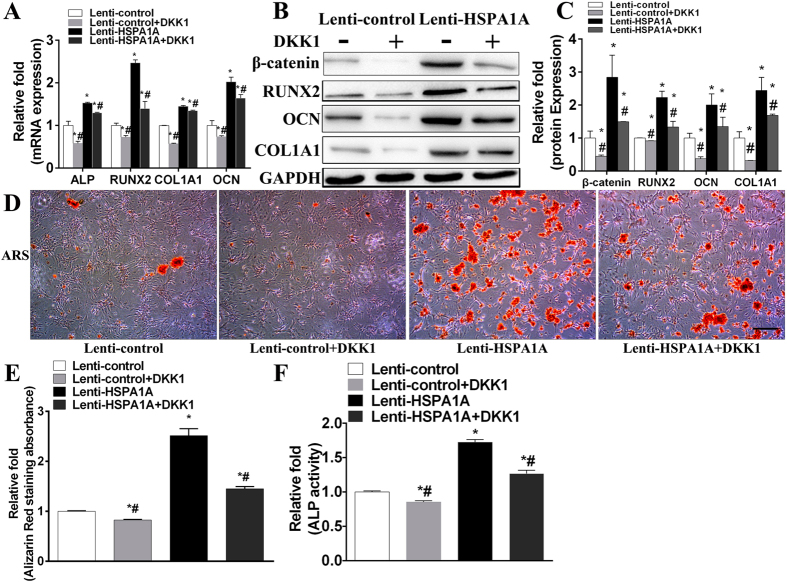
The increased osteogenesis caused by *HSPA1A* overexpression could be rescued partially by the addition of a Wnt/β-catenin signaling inhibitor (DKK1). (**A**) The expression of ALP, RUNX2, OCN, and COL1A1 mRNA in the lenti-control, lenti-control + DKK1, lenti-*HSPA1A*, and lenti-*HSPA1A* + DKK1 groups were determined by qPCR. The mRNA expression levels were normalized to that of 18S ribosomal RNA. (**B,C**) The expression of β-catenin, RUNX2, OCN, and COL1A1 in the lenti-control, lenti-control + DKK1, lenti-*HSPA1A*, and lenti-*HSPA1A* + DKK1 groups were determined by Western blot analysis. The protein expression levels were normalized to that of GAPDH. (**D**) Alizarin red staining in the lenti-control, lenti-control + DKK1, lenti-*HSPA1A*, and lenti-*HSPA1A* + DKK1 groups at day 28 of osteogenic differentiation. Scale bar = 200 μm (**E**) Alizarin red staining area determined by measuring the absorbance at 560 nm. (**F**) ALP activity in the lenti-control, lenti-control + DKK1, lenti-*HSPA1A*, and lenti-*HSPA1A* + DKK1 groups at day 14 of osteogenic differentiation. The data are expressed as means ± standard deviation of three independent experiments, and one of three independent experiments is shown. *P < 0.05 vs. the lenti-control group. ^#^*P* < 0.05 vs. the lenti-*HSPA1A* group.

To verify the involvement of the Wnt/β-catenin signaling pathway, we evaluated the inhibitory effect of this pathway on osteogenesis in the *HSPA1A*-overexpressing group. After the addition of DKK1 for 1 h, osteo-specific genes and proteins were examined. As shown in Fig. 5, lower expression levels of the osteo-specific genes ALP, RUNX2, OCN, and COL1A1 were identified in the inhibitor-treated cells (lenti-*HSPA1A*+DKK1) than in the cells with *HSPA1A* overexpression alone.

Moreover, ALP activity detection demonstrated higher ALP activity in MSCs overexpressing *HSPA1A* than in the lenti-*HSPA1A*+DKK1 group (Fig. 5F). There was also less matrix mineralization at day 28 of osteogenic differentiation in the lenti-*HSPA1A*+DKK1 group than in rBMSCs overexpressing *HSPA1A* (Fig. 5D,E).

### A sheet of rBMSCs overexpressing *HSPA1A* accelerated bone fracture healing in a rat tibial fracture model

**Figure 6:**
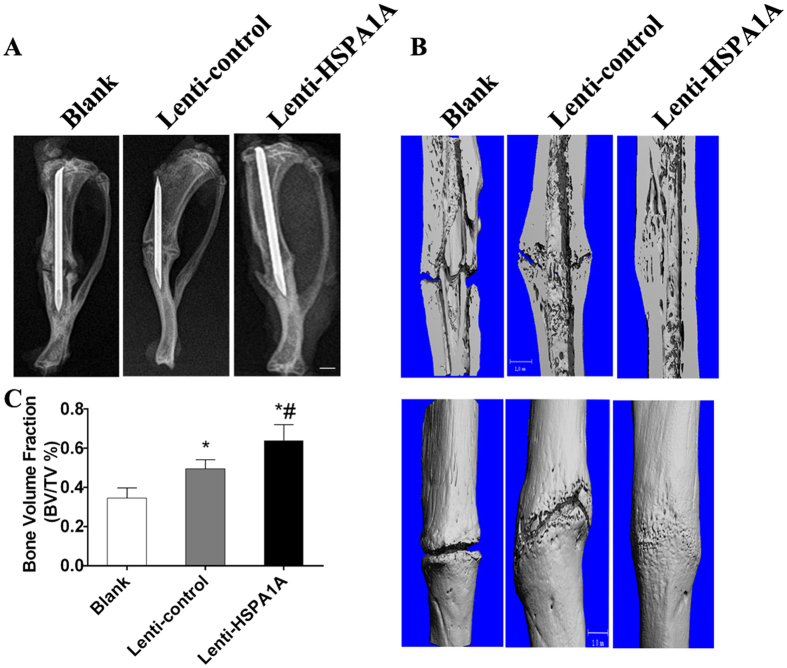
Radiographic and micro-computed tomography (CT) analyses of the fracture site at 8 weeks after surgery in each group. (**A**) Radiographic analysis of the fracture sites at 8 weeks after surgery in each group. (**B,C**) Micro-CT images of the fracture sites in each group at 8 weeks after surgery. Scale bar = 1 mm.

Radiographs taken at 8 weeks showed that the cortical gap was clearly present in the blank group. In the lenti-control group, this gap was obscure, and more bridging callus formation was evident at the fracture site compared with the blank group. In the rBMSCs overexpressing *HSPA1A*, the gap had disappeared (Fig. 6A).

The results of micro-CT indicated significantly more bone formation in the rBMSCs overexpressing *HSPA1A* than in the lenti-control group; moreover, among the three groups, the gap was widest in the blank group (Fig. 6B). Quantitatively, the fractures treated with a sheet of rBMSCs overexpressing *HSPA1A* displayed a significant increase in the BV/TV compared with those of the other two groups (Fig. 6C).

**Figure 7:**
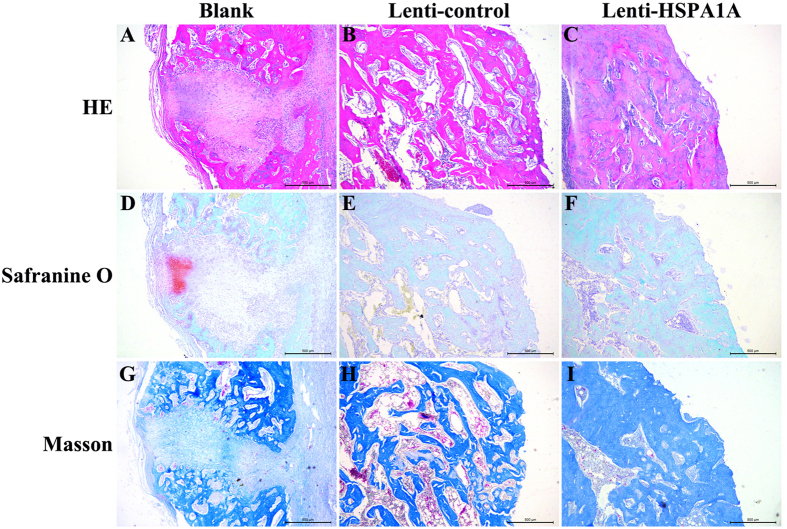
Histological evaluation of the fracture site 8 weeks after surgery in each group. (**A–C**) HE staining. Scale bar = 500 μm (**D–F**) Safranin O and fast green staining. Scale bar = 500 μm (**G-I**) Masson staining. Scale bar = 500 μm.

Histological analysis showed that the gaps were filled with fibrous tissue and a few chondrocytes; moreover, no bridging bone formation at the fracture site was observed in the blank group (Fig. 7A,D). In the lenti-control group, a thick callus consisting of newly formed woven bone tissue was detected at the fracture site, and the callus was undergoing remodeling (Fig. 7B,E). In the rBMSCs overexpressing *HSPA1A*, the fracture sites were sealed, and the remodeling of the callus was almost complete, indicating bony healing of the fracture (Fig. 7C,F).

**Figure 8:**
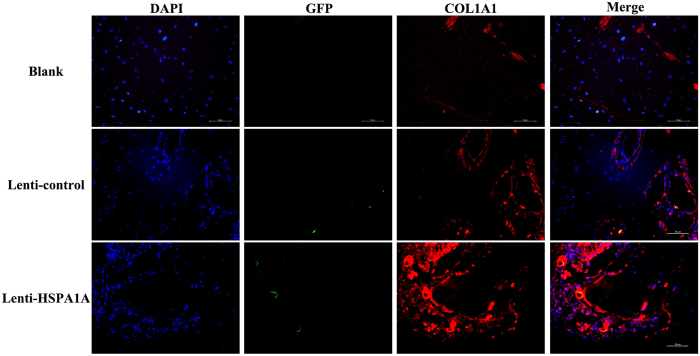
Immunofluorescence histological staining 8 weeks after surgery in each group. The nuclei were stained by DAPI with blue fluorescence. COL1A1 protein was stained with red fluorescence. Scale bar = 50 μm.

Immunofluorescence analysis showed that significantly more COL1A1 appeared at the fracture site in the rBMSCs overexpressing *HSPA1A* than in the other two groups. The lowest expression of COL1A1 was detected in the blank group. Sporadic fluorescence of GFP was found in the lenti-control group and in the rBMSCs overexpressing *HSPA1A* (Fig. 8).

## Discussion

To our knowledge, this is the first study to investigate the effects of *HSPA1A* on MSC osteogenic differentiation. We found that endogenous expression of *HSPA1A* was upregulated during osteogenesis in rBMSCs. Moreover, according to the results of previous studies, *HSPA1A* is highly expressed in new bone-healing areas^11^,^12^. Thereupon, we want to use an overexpression of *HSPA1A* strategy to accelerate osteogenesis of MSCs. We found that *HSPA1A* overexpression promoted osteogenic differentiation of rBMSCs partly via the Wnt/β-catenin signaling pathway *in vitro*. Moreover, a sheet of rBMSCs overexpressing *HSPA1A* accelerated bone fracture healing in rat fracture models. These findings indicate that *HSPA1A* overexpression enhances osteogenic differentiation of rBMSCs, at least partly through activation of the Wnt/β-catenin signaling pathway.

Mounting evidence has revealed that increased expression of *HSPA1A* is closely associated with osteogenesis. Tiffee *et al*. found that HSC70 was localized in the osteoblasts lining new bone in the primary spongiosa, which might have biosynthetic functions within osteoblasts^12^. Continuous exposure to mild heat stress (39–41 °C) significantly enhanced the ability of BMSCs to form mineralizing nodules, which were associated with *HSPA1A* constitutive expression^10^. Aisha *et al*. revealed that chaperone proteins, including HSC70, stabilized mRNA transcripts and enhanced ALP and OCN gene transcription^29^. Likewise, our results demonstrated that the expression of *HSPA1A* plays a positive role in osteogenic differentiation. We found that *HSPA1A* was upregulated during osteogenesis in rBMSCs. RUNX2 is a master transcription factor involved in osteogenic differentiation^30^. In our study, its expression was significantly increased following overexpression of *HSPA1A*. The levels of an early marker of osteogenic differentiation (ALP) and late markers of osteogenic differentiation (OCN, COL1A1) were also increased due to *HSPA1A* overexpression. In addition, this overexpression significantly enhanced mineral deposition. Meanwhile, *HSPA1A* overexpression had no adverse effects on the metabolic activity or cell proliferation of rBMSCs. These results indicate that *HSPA1A* overexpression promoted the osteogenic differentiation of rBMSCs *in vitro*.

A previous study reported crosstalk between HSC70 and Wnt/β-catenin signaling in stem cells; that is, *HSPA1A* upregulation was closely related to activation of the Wnt/β-catenin signaling pathway^20^. Wnt/β-catenin signaling is an essential pathway in the osteogenic differentiation of MSCs^14^,^15^. Wnt signaling results in cellular accumulation of Wnt/β-catenin, followed by nuclear translocation of β-catenin and activation of target genes^31^. In this study, we detected higher expression of β-catenin following overexpression of *HSPA1A* during osteogenesis. Moreover, higher β-catenin accumulation was observed, the majority of which was in the nucleus upon *HSPA1A* overexpression, suggesting that *HSPA1A* overexpression activates β-catenin-mediated transcription. Meanwhile, lower levels of DKK1 and SOST were observed, which inhibited Wnt/β-catenin by binding to the third β-propeller domain of LRP5/6 via interference of Wnt/LRP/Fz trimolecular complex formation^18^. Furthermore, the increased osteogenesis of MSCs by *HSPA1A* overexpression was rescued partly by an inhibitor of Wnt/β-catenin (DKK1). These findings indicate that overexpression of *HSPA1A* regulates the Wnt/β-catenin signaling pathway during osteogenesis of MSCs.

It has been demonstrated that MSC sheets can enhance bone regeneration^26^,^32^,^33^. Cultured cells can be harvested as intact sheets, thereby decreasing cell damage and maintaining the majority of the extracellular matrix, which can attach to host tissues and fracture sites, covering the surface with minimal cell loss^34^. In our study, the use of a rBMSC sheet promoted bone formation in a rat tibial fracture model. Better bone healing was also observed when rBMSCs overexpressing *HSPA1A* were present in the sheet. Additionally, these *HSPA1A*-overexpressing rBMSCs did not cause localized tumors or malignancies. However, there were only a few cells exhibiting GFP fluorescence in the rBMSC sheet groups, which suggested that only a fraction of the implanted rBMSCs directly differentiated into osteocytes and produced bone matrix. The majority of new bone formation arose from exogenous cells, which is consistent with previous studies^28^,^35^. Transplanted MSCs increase bone formation through the production of factors that promote osteogenic differentiation of host cells^35^. A paracrine effect might have been involved in bone healing *in vivo*, when the sheet of rBMSCs overexpressing *HSPA1A* was implanted for fracture repair. Guang *et al*. revealed that HSC70 plays a significant role in endothelial cell development and angiogenesis^36^. Aparna *et al*. also reported that HSC70 could bind and stabilize endogenous AU-rich element-containing mRNAs encoding vascular endothelial growth factor^7^. These studies indicated that HSC70 might be closely related to vasculogenesis and neovascularization of endothelial cells, which could play an important role in bone healing.

The present study has some limitations. First, although we indicated that *HSPA1A* overexpression mediates the Wnt/β-catenin signaling pathway to promote osteogenic differentiation of rBMSCs, it is probably involved in the activation of other signaling pathways as well. Second, the mechanisms of bone healing acceleration using a sheet of rBMSCs overexpressing *HSPA1A* have not been clarified completely, and thus further studies are needed.

## Conclusion

Based on our data, we found that *HSPA1A* overexpression enhanced osteogenic differentiation of rBMSCs, partly through activation of the Wnt/β-catenin signaling pathway. The use of *HSPA1A*-overexpressing MSCs to enhance fracture repair has potential as a new MSC-based therapy.

## Additional Information

**How to cite this article**: Zhang, W. *et al*. Overexpression of *HSPA1A* enhances the osteogenic differentiation of bone marrow mesenchymal stem cells via activation of the Wnt/β-catenin signaling pathway. *Sci. Rep.* **6**, 27622; doi: 10.1038/srep27622 (2016).

## Supplementary Material

Supplementary Information

## Figures and Tables

**Table 1:** Sequences of primers for quantitative real-time polymerase chain reaction.

Gene Name	Forward primer sequence (5′ to 3′)	Reverse primer sequence (5′ to 3′)
ALP	TCCGTGGGTCGGATTCCT	GCCGGCCCAAGAGAGAA
OCN	GAGCTGCCCTGCACTGGGTG	TGGCCCCAGACCTCTTCCCG
RUNX2	CCGATGGGACCGTGGTT	CAGCAGAGGCATTTCGTAGCT
COL1A1	CATCGGTGGTACTAAC	CTGGATCATATTGCACA
β-catenin	CTTACGGCAATCAGGAAAGC	TAGAGCAGACAGACAGCACCTT
DKK1	GCTTGGTGCATACCTGACCT	AAGGGCAAGAAGGCTCTGTC
SOST	ACTCGGACACGTCTTTGGTG	GTACATGCAGCCTTCGTTGC
*HSPA1A*	GGCCTTGAGGACTTTGGGTTA	TGGGAATGCAAAGCACACG
18S	CAGACAAATCGCTCCACCAA	TTGACGGAAGGGCACCA

Abbreviations: ALP, alkaline phosphatase; COL1A1, type I collagen; DKK1, Dickkopf-1; *HSPA1A*, Heat shock protein family A member 1A; SOST, sclerostin; OCN, osteocalcin; RUNX2, runt-related transcription factor 2; 18S, 18S ribosomal RNA.
