# Supplementary material for: Overexpression of HSPA1A enhances the osteogenic differentiation of bone marrow mesenchymal stem cells via activation of the Wnt/β-catenin signaling pathway
Source: Sci Rep. 2016 Jun 9;6:27622. doi: 10.1038/srep27622 (PMC4899801; doi:10.1038/srep27622)

Title: Overexpression of *HSPA1A* enhances the osteogenic differentiation of bone marrow mesenchymal stem cells via activation of the Wnt/β-catenin signaling pathway

Author names: Wei Zhang, MD[1*], Deting Xue, MD[1*], Houfa Yin, MD[2], Shengdong Wang, MD[1], Chao Li, MD[3], Erman Chen, MD[1], Dongcai Hu, MD[1], Yiqing Tao, MD[1], Jiawei Yu, MD[4], Qiang Zheng, MD[1], Xiang Gao, MD[1], Zhijun Pan, MD[1#]

The affiliation and address of the authors:

1- Department of Orthopedics, Second Affiliated Hospital, School of Medicine, Zhejiang University, 310009, Hangzhou, China.

2- Eye Center, Second Affiliated Hospital, School of Medicine, Zhejiang University, 310009, Hangzhou, China.

3- Department of Surgery, Second Affiliated Hospital of Zhejiang University School of Medicine, 310009, Hangzhou, Zhejiang Province, China.

4- Department of Orthopedics, Zhuji People's Hospital of Zhejiang Province, Zhuji, Zhejiang Province, China.

[#]**Corresponding author:**

Zhijun Pan, Tel: 0086-0571-87783567, E-mail: panzhijunzw@163.com

Supplemental material legends

Supplemental material 1: Lentiviral overexpresseding HSPA1A particles (Lenti-HSPA1A)

Supplemental material 2: Cell sheet preparation

Supplemental material 3: Animal model

Supplemental material 1: lentiviral vector

Vector Summary

**Vector ID**	VB150330-10009
**Vector Name (official)**	pLV[Exp]-Puro-CMV>rHspa1a[NM_031971.2]:IRES:EGFP
**Date Created (Pacific Time)**	2015-03-30
**Size**	11145 bp
**Vector Type**	Lentivirus gene expression vector (3rd generation)
**Inserted Promoter**	CMV
**Inserted ORF**	rHspa1a[NM_031971.2], EGFP
**Inserted Linker**	IRES
**Inserted Marker**	Puro
**Copy Number**	High
**Bacterial Resistance**	Ampicillin
**Cloning Host**	Stbl3

User Annotation of Vector

**Vector alias (from user)**	*None*
**Comment (from user)**	*None*

Vector Map

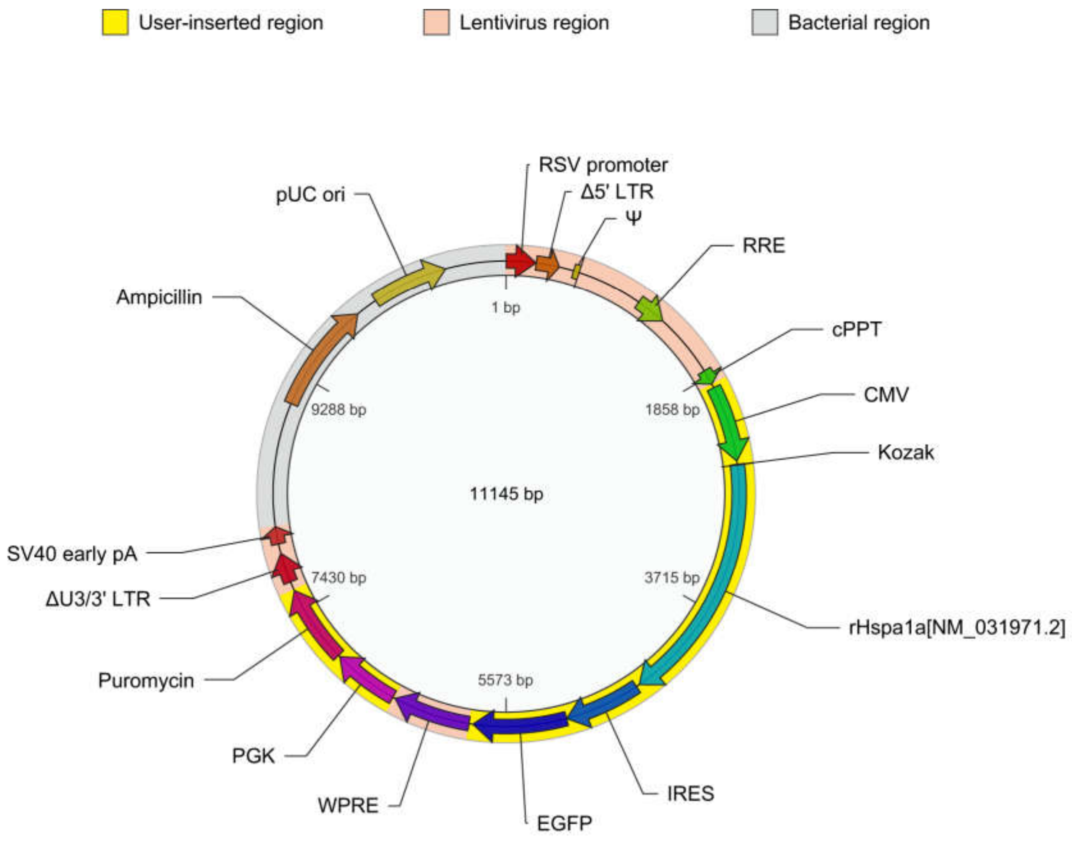

Vector Components

Component Name	Nucleotide Position	Full Name	Description
RSV promoter	**1-229**	Rous sarcoma virus (RSV) enhancer/promoter	Allows Tat-independent production of viral mRNA.
Δ5' LTR	230-410	HIV-1 truncated 5' LTR	Permits viral packaging and reverse transcription of the viral mRNA.
Ψ	**521-565**	HIV-1 psi packaging signal	Allows viral packaging.
RRE	1075-1308	HIV-1 Rev response element	Permits Rev-dependent nuclear export of unspliced viral mRNA.
cPPT	**1803-1920**	Central polypurine tract	Facilitates the nuclear import of HIV-1 cDNA through a central DNA flap.
CMV	1950-2538	CMV	Component entered by user
Kozak	**2563-2568**	Kozak	Component entered by user
rHspa1a[NM_031971.2]	2569-4494	rHspa1a[NM_031971.2]	Component entered by user
IRES	**4519-5106**	IRES	Component entered by user
EGFP	5107-5826	EGFP	Component entered by user
WPRE	**5856-6453**	Woodchuck hepatitis virus posttranscriptional regulatory element	Facilitates effective transcription termination at the 3' LTR.
PGK	6472-6982	Mouse phosphoglycerate kinase promoter	Allows high-level expression of the selection marker in mammalian cell lines.
Puromycin	**6995-7594**	Puromycin resistance gene	Permits selection of stably transduced mammalian cell lines.
ΔU3/3' LTR	7666-7900	HIV-1 truncated 3' LTR	Allows viral packaging but self-inactivates the 5' LTR for biosafety purposes. The element also contains a polyadenylation signal for transcription termination and polyadenylation of mRNA in transduced cells.
SV40 early pA	**7972-8106**	SV40 early polyadenation signal	Allows transcription termination and polyadenylation of mRNA.
Ampicillin	9061-9921	Ampicillin resistance gene	Allows selection of the plasmid in E.coli.
pUC ori	**10091-10679**	pUC origin of replication	Permits high-copy replication and maintenance in E.coli.

Note: (c) denotes complementary strand.

User Annotation of Vector Components

Component Name	Comment by User
CMV	*None*
rHspa1a[NM_031971.2]	*None*
IRES	*None*
EGFP	*None*
Puro	*None*

Supplemental material 2: Cell sheet preparation

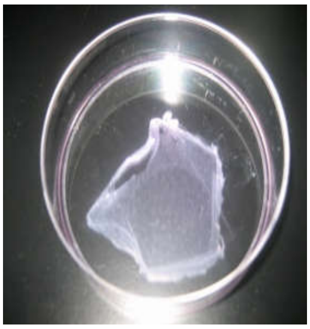

Supplemental material 3: Animal model

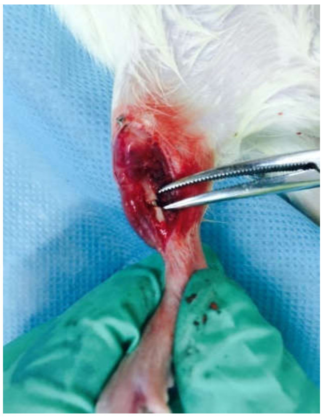

Supplement: Supplementary Information [file srep27622-s1.pdf]
